# Seroepidemiological Prevalence of Hepatitis B and C Virus Coinfection: A Large-Scale Retrospective Cohort Study in Istanbul

**DOI:** 10.3390/diagnostics16182895

**Published:** 2026-09-09

**Authors:** Arat Hulikyan, Hayriye Kirkoyun Uysal, Gizem Dagci, Bilger Cavus, Sebahattin Kaymakoglu, Murat Hakan Kir, Arif Atahan Cagatay, Derya Sevimli Saydan, Mehmet Demirci, Ali Agacfidan, Mustafa Onel

**Affiliations:** 1 Department of Medical Microbiology, Istanbul Faculty of Medicine, Istanbul University, Istanbul 34452, Turkey; arathulikyan@hotmail.com (A.H.); hkirkoyun@yahoo.com (H.K.U.); deryasevimli1@gmail.com (D.S.S.); aagacfidan@hotmail.com (A.A.); 2 Department of Medical Microbiology, Institute of Graduate Studies in Health Sciences, Istanbul University, Istanbul 34452, Turkey; 3 Division of Gastroenterology, Department of Internal Medicine, Istanbul Faculty of Medicine, Istanbul University, Istanbul 34452, Turkey; gizem.dagci@istanbul.edu.tr (G.D.); bilger.cavus@istanbul.edu.tr (B.C.); sabahattin.kaymakoglu@istanbul.edu.tr (S.K.); 4 Department of Infectious Diseases and Clinical Microbiology, Istanbul Faculty of Medicine, Istanbul University, Istanbul 34452, Turkey; murathakankir1@istanbul.edu.tr (M.H.K.); acagatay@istanbul.edu.tr (A.A.C.); 5 Department of Medical Microbiology, Faculty of Medicine, Kırklareli University, Kırklareli 39100, Turkey; mehmetdemirci@klu.edu.tr

**Keywords:** hepatitis B, hepatitis C, coinfection, retrospective cohort study, seroprevalence, viral hepatitis

## Abstract

**Background/Objectives**: Hepatitis B (HBV) and hepatitis C (HCV) coinfection leads to the progression of liver disease and results in clinically serious outcomes. Continuous monitoring of epidemiological data is crucial for managing the regional disease burden. This study investigates the current seroprevalence of HBV and HCV, the prevalence of coinfection, and the rates of active replication in Istanbul. **Methods**: This retrospective analysis included 7131 patients evaluated at the Infectious Diseases and Gastroenterohepatology clinics between 2021 and 2025. Initial serological screening for viral markers was performed using routine chemiluminescent immunoassays. Active viral replication was subsequently confirmed and quantified via real-time polymerase chain reaction (PCR). **Results**: Active HBV replication was significantly more frequent among patients in the Gastroenterohepatology clinic (20.47%) compared to those in Infectious Diseases (5.36%). Active HCV infections were rare in both departments (0.25% and 0.04%, respectively). Simultaneous HBV and HCV coinfection was detected in only 17 patients (0.24%). Demographic evaluation demonstrated that HBV marker positivity was more prevalent in male patients and individuals over 40 years of age. **Conclusions**: HBV seroprevalence remains substantially higher than HCV in this cohort. The high rate of active HBV replication in gastroenterohepatology settings indicates a concentrated burden of active disease in these specialized clinics. These distribution patterns emphasize the necessity of routine, targeted HBV screening for older male demographics to facilitate early clinical intervention.

## 1. Introduction

Viral hepatitis, primarily caused by the hepatitis B virus (HBV) and hepatitis C virus (HCV), remains a major public health challenge and a leading cause of global morbidity and mortality [1]. The World Health Organization (WHO) estimates that 254 million people currently live with chronic HBV, while 50 million have chronic HCV infections [2]. Both hepatotropic viruses are primary drivers of progressive liver disease. Chronic infection drives sustained hepatic inflammation and fibrogenesis, which can ultimately progress to end-stage liver disease, cirrhosis, and hepatocellular carcinoma (HCC) [3,4].

Although prophylactic vaccines have reduced the incidence of new HBV infections and direct-acting antivirals (DAAs) provide high cure rates for HCV, the global burden of chronic cases remains high due to asymptomatic progression and delayed diagnosis [5].

HBV is primarily transmitted through perinatal, parenteral, and sexual routes, currently affecting an estimated 254 million chronic carriers worldwide [2,6]. Prevention strategies heavily depend on universal infant vaccination programs, alongside the administration of immunoglobulins and antenatal antivirals for mothers with high viral loads [7]. In clinical practice, long-term virological suppression is typically achieved using nucleos(t)ide analogues. Historically considered a low-to-intermediate endemicity region, Turkey reports a national HBV prevalence ranging between 4.0% and 4.6% [6,8]. Although marked regional variations exist between the eastern and western parts of the country, overall prevalence rates have been steadily declining following the successful implementation of the national immunization program in 1998 [8,9].

Conversely, HCV is predominantly transmitted via parenteral routes, most notably through injection drug use or unscreened blood products. While no effective prophylactic vaccine currently exists for HCV, the advent of DAAs has revolutionized disease management, yielding virological cure rates that exceed 95% [10]. Globally, approximately 50 million individuals are chronically infected [2]. Turkey is classified as a low-endemicity region for HCV, with general anti-HCV seroprevalence fluctuating between 1.2% and 4.0%, and dropping significantly below 1.0% among voluntary blood donors [11].

Beyond progressive hepatic injury, both hepatotropic viruses are deeply implicated in a wide spectrum of severe extrahepatic manifestations. Chronic HBV infection is clinically associated with serum sickness-like syndrome, polyarteritis nodosa, glomerulonephritis, and specific dermatologic presentations, which are frequently reported in Turkish cohorts [12]. Concurrently, chronic HCV exhibits an even broader extrahepatic profile. Mixed cryoglobulinemia is observed in up to 50% of infected individuals, alongside other significant immune-mediated conditions such as Sjögren’s syndrome, autoimmune thyroiditis, porphyria cutanea tarda, insulin resistance, and an elevated risk of B-cell non-Hodgkin lymphoma, collectively impacting 38% to 76% of patients over the course of their infection [13,14].

Since HBV and HCV share parenteral, sexual, and vertical transmission routes, coinfection occurs frequently, particularly in high-endemicity areas and specific risk groups [15]. Compared to monoinfection, coinfected patients experience accelerated liver disease progression, a higher incidence of cirrhosis, and an elevated risk of HCC [16]. The interplay between these viruses often involves viral interference, where one virus suppresses the replication of the other, complicating diagnosis and treatment [17]. Coinfection prevalence is highly variable, ranging from 1% to 15% in general clinical series, yet it soars significantly among high-risk demographics, including people who inject drugs and HIV-positive cohorts [18,19]. Despite the accelerated clinical trajectory and severe outcomes associated with dual infection, comprehensive population-level data characterizing coinfection dynamics remain scarce in Turkey. Therefore, the primary aim of this study was to elucidate the current seroepidemiological prevalence of HBV, HCV, and their coinfection among a 7131-patient cohort evaluated at a major tertiary referral center in Istanbul between January 2021 and January 2025. What distinguishes this research from previous Turkish studies which largely relied on smaller samples, single high-risk demographics, older sampling periods, or non-metropolitan centers is that it offers one of the largest and most up-to-date coinfection-focused cohorts drawn from a diverse metropolitan catchment area in the post-DAA era.

## 2. Materials and Methods

### 2.1. Study Design and Patient Population

This cohort study was conducted at Istanbul University, Istanbul Faculty of Medicine. The study population included 7131 patients evaluated at the outpatient clinics of the Department of Infectious Diseases and Clinical Microbiology, and the Department of Internal Medicine, Division of Gastroenterology between January 2021 and January 2025. Venous blood samples were collected as part of routine clinical care and virological assessment. Serum and plasma aliquots were stored at −20 °C in the Virology and Basic Immunology Laboratory of the Department of Medical Microbiology for subsequent molecular analysis. Patients under long-term active follow-up who had available viral load data (HBV DNA or HCV RNA) but lacked concurrent serological screening results during the retrospective data extraction period were classified as a distinct ’Follow-up via Viral Load Only’ group to reflect real-world clinical data without introducing selection bias. The cohort was built exclusively from retrospective virological screening records (HBsAg, anti-HCV, HBV DNA, and HCV RNA) obtained directly from the laboratory registry. Because the study did not involve a linked clinical chart review, specific variables such as treatment history (including the use of interferons, direct-acting antivirals, or nucleos(t)ide analogues), concurrent HDV or HIV testing results, and biochemical or fibrosis parameters were out of the scope of the anonymized virological data covered by the ethics approval. Furthermore, it is important to note that no direct patient questionnaires were administered. Because this was a strictly retrospective, records-based study, data concerning prior HBV vaccination status or current individual antiviral regimens were not systematically recorded in the aggregate laboratory information system and were therefore unavailable as study variables.

### 2.2. Ethical Approval

The study protocol was approved by the Clinical Research Ethics Committee of Istanbul University, Istanbul Faculty of Medicine (Approval Number: 30.04.2025-3297949). All procedures complied with the ethical standards of the institutional research committee and the 1964 Declaration of Helsinki and its later amendments. Given the retrospective design, the Ethics Committee waived the requirement for written informed consent. All patient data were anonymized prior to analysis.

### 2.3. Serological Assays

Qualitative serological screening for HBsAg and anti-HCV was performed using chemiluminescent microparticle immunoassays (CMIA) on the Abbott ARCHITECT^®^ i2000SR and Alinity^®^ i platforms (Abbott Diagnostics, Chicago, IL, USA). Viral markers were detected using FDA-approved and CE-IVD marked reagent kits according to the manufacturer’s protocols.

### 2.4. Nucleic Acid Extraction and Real-Time PCR

For molecular quantification, viral nucleic acids were isolated from the stored clinical specimens using the QIAsymphony^®^ SP system (QIAGEN, Hilden, Germany). Following extraction, HBV DNA and HCV RNA levels were quantified using real-time polymerase chain reaction (qPCR) on the Cobas^®^ 6800 system (Roche Diagnostics, Mannheim, Germany). The HBV DNA assay targeted the polymerase (pol) and surface (S) gene regions, with a lower limit of quantification (LOQ) of 10 IU/mL. The HCV RNA assay targeted the 5′ untranslated region (5′ UTR), with an LOQ of 15 IU/mL.

### 2.5. Statistical Analysis

Statistical analysis was performed using SPSS version 26.0. Categorical variables, including gender, age groups (18–39, 40–64, and ≥65 years), and clinical departments, were summarized as frequencies and percentages. The Pearson Chi-square or Fisher’s exact test was used to compare categorical variables. All tests were two-tailed, and *p* < 0.05 was considered statistically significant.

## 3. Results

### 3.1. Demographic Characteristics of the Study Population

A total of 7131 patients with suspected viral hepatitis were evaluated retrospectively. The cohort comprised 3869 (54.26%) males and 3262 (45.74%) females. The largest proportion of patients fell within the 40–64 age group for both males (50.14%) and females (56.53%). Among males, the 18–39 and ≥65 age groups accounted for 36.11% and 13.76%, respectively. Overall, 2406 patients (33.74%) presented to the Infectious Diseases and Clinical Microbiology clinic, and 4725 (66.26%) to the Gastroenterohepatology clinic (Table 1). Males were predominant in both clinics (55.90% and 53.42%, respectively) (Table 2).

### 3.2. Prevalence of HBV Markers and Active Replication

HBsAg positivity was detected in 1571 patients (22.03%) across the cohort. Active viral replication, defined as concurrent HBsAg and HBV DNA positivity, was identified in 129 patients (5.36%) in the Infectious Diseases clinic and 967 patients (20.47%) in the Gastroenterohepatology clinic (*p* < 0.001). Additionally, a specific subgroup of registered chronic hepatitis patients under active follow-up presented with available viral load data but lacked concurrent serological marker results in the hospital registry system. The proportion of patients classified within this ’Follow-up via Viral Load Only’ group out of the total cohort was higher in the Gastroenterohepatology clinic compared to the Infectious Diseases clinic (6.77% vs. 4.77%). No significant difference was observed between genders regarding concurrent HBsAg and HBV DNA positivity (*p* = 0.538). (Table 3).

### 3.3. Prevalence of HCV Markers

Indicators for HCV infection were lower than those for HBV. Concurrent anti-HCV and HCV RNA positivity was detected in 6 patients (0.25%) in the Infectious Diseases clinic and 2 patients (0.04%) in the Gastroenterohepatology clinic. The proportion of anti-HCV positive but HCV RNA negative cases was higher in the Gastroenterohepatology clinic (0.80%) compared to the Infectious Diseases clinic (0.29%) (*p* = 0.0096). This serological profile was also more prevalent in females (*p* = 0.0047). Rates for the HCV ’Follow-up via Viral Load Only’ group did not differ significantly between the clinics (*p* = 0.973) or genders (*p* = 0.446) (Table 4).

### 3.4. HBV/HCV Coinfection Rates

HBV/HCV coinfection was rare, observed in 17 patients (0.24%). The distribution of coinfected cases did not differ significantly between the Infectious Diseases (*n* = 6, 0.25%) and Gastroenterohepatology (*n* = 11, 0.23%) clinics (*p* = 0.892), nor did it vary by gender (*p* = 0.913). The most frequent coinfection profile in the Gastroenterohepatology cohort was HBsAg (+)/anti-HCV (+)/HBV DNA (+)/HCV RNA (−), observed in 11 cases (Table 5).

### 3.5. Temporal Trends in Virological Prevalence (2021–2025)

**Figure 1:**
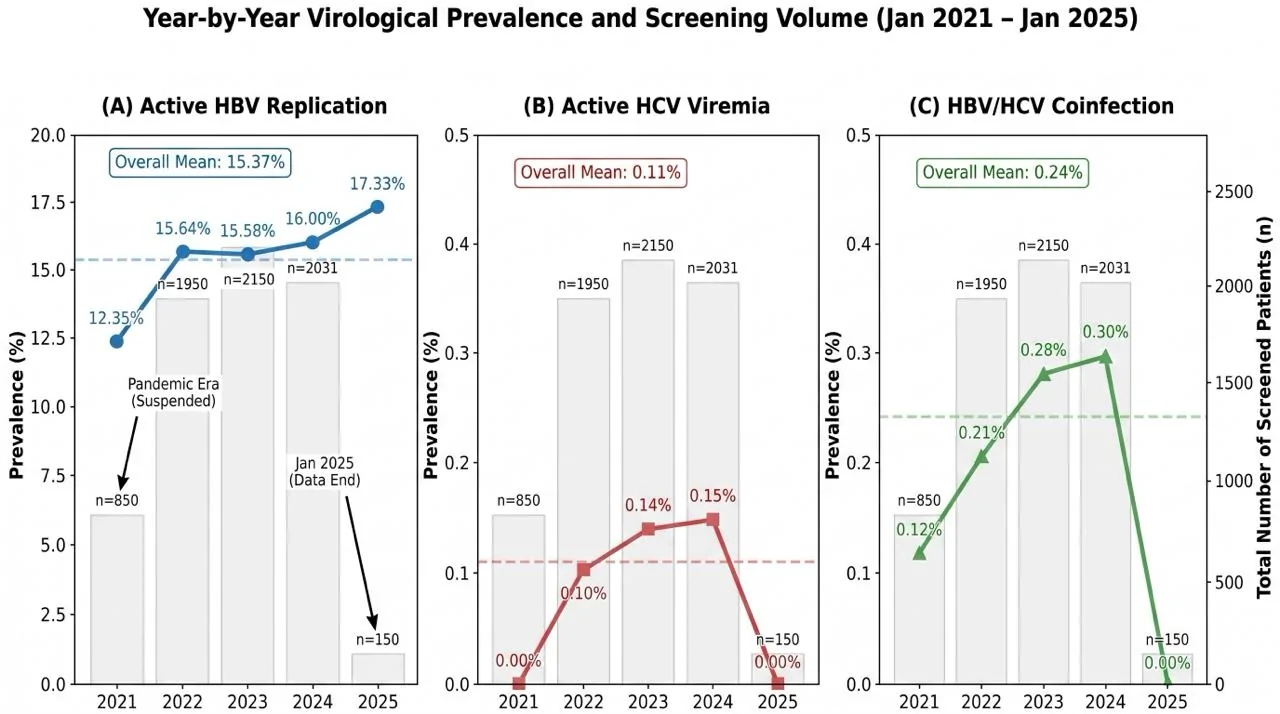
Year-by-year virological prevalence and total screening volume from January 2021 to January 2025. (**A**) Active HBV replication rates. (**B**) Active HCV viremia rates. (**C**) HBV/HCV coinfection rates. Gray bars represent the total number of screened patients (*n*) per year.

To assess longitudinal changes in infection rates, a year-by-year trend analysis was conducted across the study period (Figure 1). Total screening volumes were notably lower in 2021 (*n* = 850), reflecting the clinical disruptions and suspended routine screenings during the COVID-19 pandemic era. Screening volumes subsequently stabilized between 1950 and 2150 patients annually from 2022 to 2024. The prevalence of active HBV replication demonstrated a steady upward trajectory, rising from 12.35% in 2021 to 16.00% in 2024, and peaking at 17.33% in the partial dataset of January 2025. Conversely, active HCV viremia remained consistently negligible, peaking at merely 0.15% in 2024. HBV/HCV coinfection rates followed a similarly low-level pattern, incrementally increasing from 0.12% in 2021 to 0.30% in 2024 before dropping in the final partial year.

## 4. Discussion

In this cohort of 7131 patients, the overall prevalence of HBV/HCV coinfection was 0.24% (*n* = 17). Although HBV/HCV coinfection accelerates fibrogenesis, cirrhosis, and HCC compared to monoinfection [20], the prevalence observed in our study falls at the lower end of the global spectrum. This aligns with regional epidemiological data, such as the coinfection rates of 0.004% to 0.273% reported in Iran by Anvari et al. [21]. Conversely, the higher prevalences documented in Taiwan (8%) and Pakistan (12.5%) highlight significant geographical variation in viral hepatitis distribution [22,23].

The prevalence of HBV in our cohort was substantially higher than that of HCV. HBsAg positivity reached 22.03%, significantly exceeding the 0.8% reported by Eldeen et al. in an Egyptian cohort [24]. Active HBV replication (concurrent HBsAg and HBV DNA positivity) was more frequent in the Gastroenterohepatology clinic (20.47%) than in the Infectious Diseases clinic (5.36%), predominantly affecting males and individuals over 40 years of age. This age-specific distribution reflects the implementation of Turkey’s national HBV vaccination program in 1998; individuals over 40 were born before universal infant immunization, rendering them more susceptible to chronic infection [9,25]. Our cohort included a distinct subset of patients with documented viral replication but missing concurrent serological data. In routine clinical practice at major tertiary referral centers, once a patient’s HBsAg or anti-HCV positivity is firmly established, subsequent follow-up visits focus primarily on molecular quantification via qPCR to monitor treatment efficacy, frequently omitting redundant serological screenings. Categorizing these patients separately allowed us to preserve valuable molecular data and accurately reflect the real-world clinical workload of viral hepatitis management in Turkey. The low prevalence of active HCV infection in our cohort likely reflects the widespread use of DAAs and screening programs in Turkey. Virologically, HBV frequently suppresses HCV replication through viral interference [26,27]. This molecular competition may partially explain the low frequency of concurrent viremia in our coinfected patients [26]. Despite this viral suppression, uncontrolled coinfection accelerates severe hepatic complications, necessitating rigorous clinical monitoring [26,27,28].

Furthermore, our temporal trend analysis (Figure 1) revealed a sustained, high burden of active HBV replication throughout the study period, contrasting starkly with the consistently negligible rates of active HCV. Notably, the diminished screening volume observed in 2021 clearly reflects the operational disruptions of the COVID-19 pandemic, during which routine virological screenings were temporarily suspended or deferred. The subsequent stabilization of screening volumes and the incremental rise in active HBV prevalence underscore the persistent need for robust outpatient screening and linkage to care in the post-pandemic era. Despite a low overall coinfection rate, clinical risk remains concentrated in vulnerable populations. Globally, high-risk groups for HBV and HCV transmission include people who inject drugs (PWID), HIV-positive individuals, incarcerated populations, and patients receiving frequent blood transfusions or hemodialysis [29,30,31,32]. As a major tertiary referral center, our institution evaluates a higher proportion of complex cases, transplant candidates, and individuals with multiple risk factors than the general population. Therefore, targeted periodic hepatitis screening in these high-risk cohorts is essential [28,33]. Early diagnosis facilitates timely therapeutic intervention to prevent cirrhosis and HCC, while also limiting viral transmission [28]. In a study by Oltmanns et al., the impact of virological dominance patterns on the cellular inflammatory milieu was investigated in patients with HBV and HCV coinfection [34]. The research demonstrated that HBV dominance influenced the expression of 58 different soluble immune mediators, specifically activating the JAK-STAT pathway and the Th17/IL-17 signaling axis [34]. The high rates of active HBV replication detected in our cohort, particularly in the Gastroenterohepatology clinic, align with these cellular mechanisms. This supports the potential of HBV activity to enhance liver fibrogenesis and progressive hepatic damage.

Tseng and colleagues explored the suppressive effect of HCV viremia on HBV biomarkers in patients with HBV/HCV coinfection [22]. The study reported that active HCV infection suppressed HBV polymerase, causing a significant decline in the serum HBV DNA, HBsAg, and HBcrAg levels while leading to an increase in HBV pgRNA levels due to an ineffective reverse transcription mechanism [22]. The extremely low rates of active HCV infection in our cohort indicate the absence of this suppressive effect of HCV on HBV, which virologically explains the high rates of active HBV replication observed among our patients. The elimination of viral interference through antiviral treatments introduces a clinical risk of HBV reactivation. In a retrospective study conducted by Park et al., the HBV reactivation rate among HBsAg-positive patients receiving HCV treatment was found to be 31.9% [35]. Reactivation developed more frequently (53.8%) and within a shorter period (median 6.4 months), particularly in patients who received DAA therapy following prior interferon treatment failure [35]. These data indicate that the successful clearance of HCV rapidly removes the suppression of HBV replication, proving that the need for targeted screening and continuous clinical monitoring emphasized in our study is critical, especially for patients receiving modern DAA therapies. The demographic and genotypic characteristics of coinfections vary across geographic regions. Research involving 2500 HCV patients in Pakistan determined an HBV coinfection prevalence of 12.5%, noting that the infection occurred significantly more often in females [23]. Furthermore, HCV genotype 3a and HBV genotype D were identified as the most frequent combination, and the presence of HBV genotype D was specifically associated with high viral loads and increased liver damage (elevated ALT levels) [23]. Conversely, the predominance of the male gender and individuals over 40 years of age in our Turkish cohort highlights the determining role of socio-demographic factors and national vaccination programs on virological distribution. The clinical burden of viral hepatitis coinfections becomes even more pronounced in specific and vulnerable populations. In a study conducted by García and colleagues on HIV-positive pregnant women in Venezuela, the HBV coinfection rate was reported as 9.3%, while the HCV coinfection rate was 3.2% [36]. These high rates were directly linked to inadequate prenatal care, economic limitations, and insufficient serological screening capabilities [36]. Although our study was conducted in a tertiary referral center, our findings regarding the vital importance of targeted periodic screening in high-risk populations parallel these international data. Effective management of viral coinfections necessitates integrated screening approaches that allow for the simultaneous detection of different viruses. Eleje et al. evaluated the burden of hepatotoxicity and maternal complications caused by infections such as HIV, HBV, HCV, and syphilis in pregnant and non-pregnant women [37]. The study emphasized that multiple test panels integrated into standard antenatal care increased the infection detection rates, reduced healthcare worker workload, and provided a cost-effective solution in low-resource settings [37]. In a comprehensive observational cohort study encompassing 1652 patients in Guizhou, China, Wang et al. investigated the prevalence of HCV/HBV coinfection and the subsequent risk of HBV reactivation following DAA therapy [38]. The investigators identified an alarmingly high HCV/HBV coinfection rate of 49.88% within their cohort yet noted that the overall cumulative incidence of HBV reactivation remained relatively low at 1.2% [38]. However, this reactivation risk escalated dramatically to 16.67% specifically in HBsAg-positive patients who did not receive prophylactic nucleos(t)ide analogue therapy, with baseline HBsAg levels exceeding 185 IU/mL serving as a significant independent predictor of viral flare [38]. Contrasting these findings with our Turkish cohort revealed a stark epidemiological and clinical dichotomy; our population demonstrated a remarkably rare overall coinfection prevalence of only 0.24%.

Nevertheless, baseline HBsAg positivity within our study reached a substantial 22.03%, and active HBV replication established an overwhelming dominance at 20.47% in the Gastroenterohepatology clinic. This immense baseline HBV burden inherently places our patients in the highest risk category for the severe virological reactivations described by Wang et al., thereby substantiating our strong recommendation for continuous clinical monitoring and targeted prophylactic interventions in HBsAg-positive individuals [38]. To elucidate the molecular mechanisms dictating these virological shifts during anti-HCV therapy, Zeng et al. provided a detailed characterization of viral interference operating at the cellular level [39]. Their research demonstrated that the active replication of HCV within hepatocytes robustly triggers host pattern recognition receptors, particularly RIG-I, MDA5, and Toll-like receptor 3 [39]. This intense activation initiates an aggressive innate immune cascade that upregulates the production of interferon (IFN) alongside crucial interferon-stimulated genes (ISGs), such as APOBEC3G, MxB, and ISG20, which actively degrade HBV covalently closed circular DNA (cccDNA) and physically suppress HBV replication [39]. The abrupt pharmacological eradication of HCV via DAA therapy rapidly dismantles this IFN-mediated shield, providing a definitive molecular rationale for the sudden reactivation of HBV [39]. This mechanistic framework flawlessly accounts for the striking clinical asymmetry observed in our 7131-patient cohort. Because active HCV infection is almost completely eradicated in our population (documented at merely 0.04% to 0.25%), the HCV-induced IFN suppression has fundamentally collapsed. The absence of this viral interference perfectly explains the uninhibited and exceptionally high rates of active HBV replication (up to 20.47%) that we recorded, confirming that the molecular void left by HCV is rapidly exploited by HBV. The exact temporal kinetics of this lost viral interference were meticulously tracked by Colombatto et al. in a cohort of HBeAg-negative patients undergoing DAA therapy [40]. By measuring serum levels of the interferon-gamma-inducible protein of 10 kDa (IP-10/CXCL10), the researchers demonstrated that the HCV-induced IFN response fades almost immediately, with IP-10 levels dropping significantly by week four of treatment in direct correlation with HCV RNA clearance [40]. Coinciding with this loss of immune pressure, the study documented a rapid and moderate increase in HBV DNA; however, this was paradoxically accompanied by a significant decline in HBsAg levels, particularly in female patients and those treated with Sofosbuvir-based regimens, pointing toward a complex, transient immunomodulatory shift mediated by natural killer cells [40]. In our study, we proactively addressed the clinical complexities of monitoring these dynamic molecular shifts by categorizing a distinct subset of patients under the ’Follow-up via Viral Load Only’ group. By identifying individuals with documented active viral replication whose redundant serological markers were omitted during routine follow-ups, our methodological design accurately reflects the real-world clinical vigilance required to capture the exact, rapid surges in HBV DNA described by Colombatto et al., ensuring that transient serological fluctuations do not mask underlying viral flares [40]. The long term oncogenic culmination of dual viral persistence was investigated by Li et al. through a comprehensive next-generation sequencing analysis of 313 virus-related hepatocellular carcinomas [41]. Their genomic profiling revealed that 55% of HBV/HCV related HCCs (HBCV-HCC) possessed clonal HBV DNA integrations that directly drove carcinogenesis via insertional mutagenesis, predominantly targeting the TERT promoter and MLL4 genes [41]. Intriguingly, the study indicated that while the presence of HCV superinfection resulted in significantly elevated somatic mutation rates in CTNNB1 and the TERT promoter, it paradoxically delayed the overall onset of tumorigenesis, leading to an older age at HCC diagnosis and more favorable postoperative outcomes compared to HBV monoinfected tumors [41]. In our clinical cohort, we similarly observed that active HBV replication and the cumulative risk of severe progressive liver disease were heavily concentrated in older demographics, specifically individuals over 40 years of age. The genomic data from Li et al. substantiates our demographic observations; even if HCV superinfection historically delayed the carcinogenic timeline in these older patients, their integrated HBV DNA remains a persistent, ticking oncogenic driver, forcefully reinforcing our conclusion that this specific aging demographic requires proactive and aggressive virological screening to prevent end-stage HCC [41]. The broader epidemiological trajectory of hepatotropic infections, particularly when superimposed with HIV, further complicates the landscape of viral hepatitis management, as demonstrated by another study from Li et al. [42]. By tracking temporal shifts in a massive cross-sectional cohort of 11,024 newly diagnosed HIV-positive subjects across China between 2015 and 2023, the researchers documented a significant national decline in both HIV/HBV (from 13.8% to 9.0%) and HIV/HCV (from 4.8% to 2.4%) coinfection rates [42]. However, their spatial and demographic analyses revealed that this decline was absent in certain geographical regions and that the risk of coinfection remained stubbornly concentrated among highly vulnerable populations, specifically individuals with low educational attainment and those engaging in intravenous drug use [42]. Although our 7131-patient study originates from a major tertiary referral center in Istanbul rather than focusing exclusively on HIV-positive networks, our epidemiological conclusions parallel these global findings. We clearly emphasized that despite a low overall coinfection rate of 0.24%, the clinical risk remains intensely concentrated in vulnerable populations, complex cases, and transplant candidates. The persistence of viral reservoirs in specific high-risk niches, as highlighted by Li et al., directly supports our assertion that periodic, targeted screening must be aggressively implemented in vulnerable groups to effectively limit viral transmission [42].

The critical role of socio-demographic determinants and health literacy in sustaining these viral reservoirs is unequivocally supported by data from sub-Saharan Africa. In a cross-sectional serosurvey conducted among 400 patients visiting hospitals in Kogi State, Nigeria, Ali et al. recorded specific prevalence rates of 3.0% for HBV, 1.0% for HCV, and 4.0% for HIV [43]. Their analysis isolated a pivotal driver of transmission: an absolute lack of knowledge regarding disease prevention and control strategies was significantly associated with heightened seropositivity across all three viral lineages [43]. Furthermore, the concentration of HBV among younger, single demographics and HCV among married cohorts illustrated how distinct behavioral practices and profound informational deficits perpetuate the endemicity of these viruses in resource-limited settings [43]. In our Turkish cohort, the demographic distribution diverged significantly, with a pronounced dominance of the male gender and individuals over 40 years of age dictating the virological burden. This contrast highlights the immense success of Turkey’s 1998 national HBV vaccination program, which effectively shielded younger generations and shifted the chronic disease burden to older, unvaccinated populations. Nevertheless, the fundamental principle aligns with Ali et al.; whether driven by a lack of health literacy or historical vaccination gaps, sociodemographic vulnerabilities dictate infection patterns, validating our call for targeted, age-specific screening protocols [43].

Despite the compounded immunosuppressive burden of multiple infections, the immuno-virologic recovery of coinfected patients can be fully preserved under optimized therapeutic conditions. Annison et al. evaluated the clinical trajectories of 500 adult HIV patients receiving highly active antiretroviral therapy (HAART) in Ghana, specifically assessing the impact of background HBV (8.4% prevalence) and HCV (0.2% prevalence) coinfections [44]. Their robust immunological data proved that the presence of chronic viral hepatitis did not significantly derail or blunt the CD4 T-cell reconstitution or the achievement of profound HIV-1 RNA viral suppression, which was successfully attained in 67.2% of the entire cohort [44]. These findings provide critical clinical reassurance that modern potent therapies are capable of overriding the pathological synergy of coinfection [44]. This perfectly echoes the clinical reality observed in our study; just as HAART effectively suppresses HIV regardless of concurrent hepatitis, the widespread use of direct-acting antivirals in Turkey has successfully suppressed HCV replication, leading to the exceptionally low concurrent viremia seen in our coinfected patients. Both studies ultimately highlight that proactive therapeutic intervention and vigilant clinical monitoring are incredibly successful at neutralizing viral threats and preventing severe clinical outcomes in complex, coinfected patients [44].

Our study has several limitations. The retrospective, single-center design may introduce selection bias, as the cohort comprises patients referred to specialist outpatient clinics rather than the general population. Specifically, the Infectious Diseases and Gastroenterohepatology clinics were purposively selected because within our institution, they serve as the designated primary referral hubs where patients with suspected or confirmed chronic viral hepatitis are routed for definitive diagnosis and treatment. However, we acknowledge that restricting sampling strictly to these two specialized clinics likely under-ascertains patients with underlying liver disease who initially present to other departments (such as internal medicine, surgery, or obstetrics). A comprehensive hospital-wide screening model in the future would yield a more complete epidemiological picture. Another major limitation of our study is its reliance on a retrospective laboratory database rather than integrated clinical charts. Consequently, we were unable to assess detailed demographic variables, specific modes of infection, or longitudinal clinical outcomes such as the progression to liver cirrhosis, hepatocellular carcinoma, or death among the coinfected and monoinfected groups. Additionally, while standard care typically involves DAA for HCV and nucleos(t)ide analogues for HBV, individual treatment histories and potential HDV or HIV coinfections were not recorded within this dataset. The relatively small number of coinfected patients further limited our ability to perform extensive comparative clinical analyses between the groups. Future prospective, multicenter, and linked-registry studies are strongly recommended to capture these critical clinical and prognostic parameters, evaluate the efficacy of active hospital-wide screening strategies, and define the molecular mechanisms of viral interference.

## 5. Conclusions

In conclusion, while HBV/HCV coinfection was rare (0.24%) within this Turkish cohort, active HBV replication remains a substantial clinical burden. The significant disparity between HBV and HCV prevalence likely reflects the success of current HCV therapies while highlighting ongoing challenges in HBV control. Given the cumulative risk of severe liver disease and HCC, proactive virological screening is essential. Targeted screening of males, individuals over 40, and defined high-risk populations is crucial to prevent end-stage liver complications and limit viral transmission.

## Figures and Tables

**Table 1:** Distribution of patients according to clinical department and gender.

Clinic/Department	Total *n* (%)	Male *n* (%)	Female *n* (%)
Infectious Diseases	2406 (33.74%)	1345 (55.90%)	1061 (44.10%)
Gastroenterohepatology	4725 (66.26%)	2524 (53.42%)	2201 (46.58%)
**Total**	**7131 (100%)**	**3869 (54.26%)**	**3262 (45.74%)**

**Table 2:** Age and gender distribution of patients according to clinical departments.

Clinic/Department	Gender	18–39 Years *n* (%)	40–64 Years *n* (%)	≥65 Years *n* (%)
Infectious Diseases	Male	486 (36.13%)	674 (50.11%)	185 (13.76%)
Infectious Diseases	Female	232 (21.87%)	601 (56.64%)	228 (21.49%)
Gastroenterohepatology	Male	911 (36.09%)	1266 (50.16%)	347 (13.75%)
Gastroenterohepatology	Female	487 (22.13%)	1243 (56.47%)	471 (21.40%)

**Table 3:** Prevalence of HBV serological and molecular markers.

Marker Profile	Inf. Dis. (Male) *n* = 1345 (%)	Inf. Dis. (Female) *n* = 1061 (%)	Gastro. (Male) *n* = 2524 (%)	Gastro. (Female) *n* = 2201 (%)	*p*-Value (Clinics)	*p*-Value (Gender)
HBsAg (+)/HBV DNA (+)	64 (4.76%)	65 (6.13%)	540 (21.39%)	427 (19.40%)	<0.001	0.538
HBsAg (+)/HBV DNA (−)	22 (1.64%)	10 (0.94%)	242 (9.58%)	184 (8.36%)	<0.001	NS
Follow-up via Viral Load Only	189 (14.05%)	151 (14.23%)	272 (10.78%)	211 (9.59%)	<0.001	NS

**Table 4:** Prevalence of HCV serological and molecular markers.

Marker Profile	Inf. Dis. (Male) *n* = 1345 (%)	Inf. Dis. (Female) *n* = 1061 (%)	Gastro. (Male) *n* = 2524 (%)	Gastro. (Female) *n* = 2201 (%)	*p*-Value (Clinics)	*p*-Value (Gender)
Anti-HCV (+)/HCV RNA (+)	3 (0.22%)	3 (0.28%)	2 (0.08%)	0 (0.00%)	0.0020	NS
Anti-HCV (+)/HCV RNA (−)	1 (0.07%)	6 (0.57%)	14 (0.55%)	24 (1.09%)	0.0096	0.0047
Follow-up via Viral Load Only	13 (0.97%)	5 (0.47%)	13 (0.52%)	22 (1.00%)	0.973	0.446

**Table 5:** Serological and molecular profiles of HBV/HCV coinfected patients.

Coinfection Profile	Inf. Dis. (Male) *n* = 1345 (%)	Inf. Dis. (Female) *n* = 1061 (%)	Gastro. (Male) *n* = 2524 (%)	Gastro. (Female) *n* = 2201 (%)	*p*-Value (Clinics)	*p*-Value (Gender)
HBsAg (+)/Anti-HCV (+)	1 (0.07%)	1 (0.09%)	0 (0.00%)	0 (0.00%)	NA	NA
HBsAg (+)/Anti-HCV (+)/HBV DNA (+)/HCV RNA (−)	2 (0.15%)	2 (0.19%)	6 (0.24%)	5 (0.23%)	0.892	0.913
**Total Coinfected Patients**	**3 (0.22%)**	**3 (0.28%)**	**6 (0.24%)**	**5 (0.23%)**	**0.892**	**0.913**

## Data Availability

The data presented in this study are available on request from the corresponding author. The data are not publicly available due to privacy and ethical restrictions.
